# The Association of OLFM4 with the Progression and Cisplatin Resistance of Head and Neck Squamous Carcinoma

**DOI:** 10.3390/curroncol32050276

**Published:** 2025-05-13

**Authors:** Xinlu He, Xi Yao, Keling Pang, Xulin Chen, Zhengbo Wei, Ying Xie

**Affiliations:** 1 Affiliated Tumor Hospital of Guangxi Medical University, Nanning 530021, China; 202220784@sr.gxmu.edu.cn (X.H.); yaoxi@sr.gxmu.edu.cn (X.Y.); 202220768@sr.gxmu.edu.cn (K.P.); 2 Key Laboratory of Early Prevention and Treatment for Regional High Frequency Tumor (Guangxi Medical University), Ministry of Education, Nanning 530021, China; 202221689@sr.gxmu.edu.cn; 3 Life Sciences Institute of Guangxi Medical University, Nanning 530021, China

**Keywords:** olfactomedin 4, ferroptosis, oxidative stress, drug resistance, cisplatin

## Abstract

Head and neck squamous cell carcinoma (HNSCC) is a highly prevalent malignant tumor globally with a poor prognosis. Despite continuous advancements in treatment modalities, the molecular mechanisms underlying its progression and chemotherapy resistance remain unclear. In previous studies, cisplatin drug induction was performed on HNSCC patient-derived tumor organoids (HNSCC-PDOs), successfully establishing a cisplatin-resistant organoid model (HNSCC-PDO^cisR^). This study conducted RNA sequencing on cisplatin-resistant HNSCC-PDO^cisR^ and their parental PDOs. Bioinformatic analysis revealed that the oncoprotein olfactomedin 4 (OLFM4) was significantly upregulated in the drug-resistant model. Combined analysis of TCGA and CPTAC databases demonstrated that OLFM4 expression correlates with poor clinical prognosis in HNSCC. In vitro cellular experiments verified that OLFM4 overexpression significantly enhanced HNSCC cell proliferation, migration, and invasion capabilities (*p* < 0.05), while OLFM4 knockdown inhibited these phenotypes. Additionally, OLFM4 was found to mediate cisplatin resistance by regulating levels of reactive oxygen species (ROS), malondialdehyde (MDA), and ferrous ions (Fe^2^⁺), suppressing cisplatin-induced oxidative stress and ferroptosis while maintaining mitochondrial membrane potential. This study confirms that OLFM4 enhances tumor cell proliferation, migration, and resistance to cisplatin-induced cell death, thereby promoting HNSCC progression. These findings suggest OLFM4 may serve as a prognostic biomarker for HNSCC and a potential therapeutic target to reverse cisplatin resistance in HNSCC.

## 1. Introduction

Head and neck squamous cell carcinoma (HNSCC) ranks as the sixth-most common malignant tumor worldwide, with approximately 700,000 new cases and 380,000 deaths reported annually [1]. This malignancy primarily originates from the mucosal epithelium of the oral cavity, larynx, pharynx, and other regions. Although treatment strategies for HNSCC, including surgery, chemotherapy, radiotherapy, targeted therapy, and immunotherapy with immune checkpoint inhibitors, have advanced significantly in recent years, the prognosis remains poor, particularly for patients with advanced-stage disease, due to high rates of local recurrence and metastasis [2]. Notably, the 5-year survival rate for HNSCC patients is only around 60%, dropping to as low as 20% for those ineligible for surgery [3]. Given these challenges, elucidating the molecular mechanisms driving HNSCC progression and identifying novel, reliable prognostic biomarkers are of critical importance. Such efforts could facilitate earlier diagnosis and the discovery of more effective therapeutic targets.

Numerous factors are closely associated with the development and poor prognosis of HNSCC, including HPV infection status, advanced clinical stage, heavy alcohol and tobacco use, genetic alterations, and incomplete treatment response. Among these, incomplete treatment response, particularly chemoresistance, is a major contributor to unfavorable outcomes in HNSCC. Currently, cisplatin (DDP) remains the most widely used chemotherapeutic agent for HNSCC, serving as a cornerstone of treatment for advanced-stage patients [4,5]. However, many patients develop cisplatin resistance during therapy, leading to disease recurrence. This acquired resistance is a primary cause of cisplatin treatment failure in HNSCC [6], yet its underlying mechanisms remain incompletely understood.

Previous studies suggest that cisplatin resistance involves multiple pathways, such as reduced intracellular cisplatin accumulation [7], enhanced DNA repair capacity in cancer cells [8,9], inactivation of cisplatin by sulfhydryl-containing molecules [8], and dysregulation of signaling pathways like PI3K/AKT and JNK [10]. Additionally, abnormal upregulation of antioxidant defenses has been implicated in cisplatin resistance across various cancers. Cisplatin exerts its cytotoxic effects by generating reactive oxygen species (ROS) to damage DNA and trigger cell death, yet cancer cells can adapt by enhancing mitochondrial respiration to fuel antioxidant mechanisms that neutralize ROS and repair DNA damage, while dysregulated redox activity disrupts ferroptosis and apoptosis pathways, enabling cells to evade cisplatin-induced cytotoxicity and thus contribute to resistance [11,12,13,14].

Identifying novel factors linked to cisplatin resistance could reveal promising therapeutic targets. Research in this field has employed diverse in vitro and in vivo models, including 2D tumor cell lines, patient-derived organoids (PDOs), and patient-derived xenografts (PDXs). Among these, PDOs have emerged as a powerful 3D ex vivo model for studying chemotherapy resistance, particularly to agents like cisplatin [15,16]. PDOs retain the genetic, epigenetic, and phenotypic heterogeneity of the original tumor, including rare resistant subclones, while preserving their 3D architecture and tumor microenvironment (TME) [17]. This enables more accurate recapitulation of TME-mediated resistance mechanisms, offering distinct advantages over conventional 2D cultures and PDXs.

To identify novel regulators of cisplatin resistance, transcriptomic profiling of cisplatin-sensitive and -resistant HNSCC PDOs was performed. This analysis led us to focus on olfactomedin 4 (OLFM4), a gene implicated in epithelial cancers, but whose role in HNSCC chemoresistance remains unclear. In the preliminary work, patient-derived organoid (PDO) models were successfully established from tumor tissues of HNSCC patients and maintained in long-term culture in vitro. Through gradual exposure to stepwise increases in cisplatin concentration, researchers induced the development of cisplatin resistance in these organoids (designated as HNSCC-PDO^cis-R^). After nearly one year of continuous culture, these organoids demonstrated significant cisplatin resistance compared to their parental counterparts. Notably, even after more than one month of drug withdrawal, the resistance index of PDO^cis-R^ remained 5.31-fold higher than that of the parental PDOs [18]. Subsequent RNA sequencing (RNA-seq) and bioinformatic analyses identified differentially expressed genes (DEGs) between HNSCC-PDO and their PDO^cis-R^ counterparts, which were prioritized for functional validation [18,19].

Building on these findings, this study aims to further elucidate the molecular mechanisms underlying cisplatin resistance and HNSCC progression using PDO models. By leveraging our preliminary data, this study aims to uncover potential therapeutic targets for this aggressive malignancy.

## 2. Materials and Methods

### 2.1. Data Acquisition and Processing

Based on the data of our previous RNA-seq study utilizing HNSCC-PDOs and HNSCC-PDO^cis-R^ [18], bioinformatic analyses were performed to screen DEGs associated with HNSCC development and cisplatin resistance. Volcano plots and heatmaps of the top 100 DEGs were generated, complemented by Venn diagram analysis of cancer-related genes (DO-enriched) and mitochondrial component genes (GO-enriched).

We extracted OLFM4-related expression data and clinical information from the Cancer Genome Atlas (TCGA) database for HNSCC. We selected 502 HNSCC patient samples and 44 adjacent normal tissue specimens based on criteria including age, sex, race, smoking history, clinical stage, and treatment history. RNA-seq gene expression data (presented as means ± SD) were analyzed alongside clinical features such as TNM stage, histological grade, patient demographics, smoking and drinking history, and radiotherapy history.

Database applications included: TIMER analysis to assess OLFM4 differential expression across tumor types; Kaplan–Meier Plotter to evaluate associations between OLFM4 expression and clinical outcomes; and the LinkedOmics LinkFinder module to analyze OLFM4-associated DEGs in TCGA-HNSCC data. Survival curves were generated based on OLFM4 expression levels (high/low), with hazard ratios (HRs) and *p*-values calculated with 95% CIs. Boxplots were created based on OLFM4 expression levels, clinical stages (AJCC 8th edition), tumor grade, gender, and body weight. Gene enrichment analysis (GSEA) identified relevant signaling pathways, while Gene Expression Profiling Interactive Analysis (GEPIA) predicted the top 50 OLFM4-correlated genes (positive/negative). STRING analysis identified 50 OLFM4-interacting proteins, with functional relationships examined through Venn diagrams and enrichment analysis.

### 2.2. Cell Culture

Human HNSCC cell lines (5-8F, CAL27, SAS, SCC-9), normal oral keratinocytes (NOKs), and patient-derived models (HNSCC-PDOs, HNSCC-PDO^cis-R^) were maintained in the Guangxi Key Laboratory for Early Prevention and Treatment of High-Incidence Tumors. The cells were cultured in DMEM (89% medium, 10% FBS, 1% penicillin) at 37 °C with 5% CO_2_.

### 2.3. Overexpression or Knockdown of OLFM4

Lentiviral constructs (Sangon Biotech, Shanghai, China) were used to generate OLFM4-overexpressing vectors (puromycin-resistant) and OLFM4-targeting shRNAs. Cells were infected (multiplicity of infection (MOI) = 30, 6 h) and selected with puromycin (2 weeks). Empty vector-infected cells served as the negative control (NC). Experimental groups were as follows. Groups of CAL27 cells included CAL27-OE-OLFM4, CAL27-OE-NC, CAL27-shOLFM4, and CAL27-shNC, and groups of 5-8F cells included 5-8F-OE-OLFM4, 5-8F-OE-NC, 5-8F-shOLFM4, and 5-8F-shNC.

### 2.4. Cell Proliferation and Cisplatin Toxicity Assay

Cell proliferation and cisplatin sensitivity were assessed in CAL27 and 5-8F cells following OLFM4 genetic manipulation using the CCK-8 kit (Biosharp, Hefei, China, BS350C), with cells seeded at 1 × 10^4^ cells/mL (100 μL/well in 96-well plates, n = 5 replicates) and cultured under standard conditions (37 °C, 5% CO_2_). Proliferation kinetics were determined on days 1, 2, 3, 4, and 5 by replacing spent medium with 100 μL CCK-8 working solution (1:5 dilution in complete medium), incubating for 2 h, and measuring optical density (OD) values at 450 nm. For cisplatin toxicity assays, initial cell density was adjusted to 1 × 10^4^ cells/well with cisplatin concentrations ranging from 0–8 μg/mL (0, 0.5, 1, 2, 4, 8 μg/mL), following identical CCK-8 processing protocols to evaluate dose-dependent cytotoxicity.

### 2.5. Cell Migration

Ibidi culture-insert assays were conducted by seeding 3.5 × 10^4^ cells/well (triplicates). At 90% confluency, inserts were removed to create standardized wounds. Cells were maintained in 2% FBS/DMEM and cultured at 37 °C in 5% CO_2_, with wound closure documented at 0, 10, 20, and 30 h. Migration rates were calculated as ((initial area − final wound area)/initial area) × 100% using ImageJ (version1.53).

### 2.6. Cell Invasion

Transwell invasion assays were performed using Matrigel-coated chambers (1:30 dilution, 100 μL/well, polymerized 2 h at 37 °C). Cells (5 × 10^4^ in 200 μL serum-free medium) were seeded in upper chambers, with 10% FBS/DMEM as chemoattractant in lower chambers. After 48 h, non-invading cells were removed and migrated cells were fixed (4% paraformaldehyde, 15 min), stained (0.1% crystal violet, 10 min), and quantified (5 random fields/chamber).

### 2.7. Real-Time Quantitative Polymerase Chain Reaction (RT-qPCR)

Total RNA was extracted from the cells using a commercial extraction kit (SevenFast, Beijing, China, SM130) according to manufacturer protocols. The steps of qPCR were as follows: initial denaturation at 95 °C for 30 s (1 cycle) and amplification at 95 °C for 5 s and at 60 °C for 30 s (40 cycles). Target-specific primer sequences are provided in Table 1. Relative gene expression was calculated using the comparative threshold cycle (2^−ΔΔCt^) method, with normalization to GAPDH.

### 2.8. Western Blotting

Cells were lysed in RIPA buffer (protease/phosphatase inhibitor (1:100)), quantified using a BCA protein assay kit (Beyotime, Shanghai, China, P0009), denatured for 10 min, and separated using 4–12% SDS-PAGE. Subsequently, proteins were transferred to nitrocellulose membranes, blocked for 15 min at room temperature, and probed with primary antibodies overnight at 4 °C, followed by incubation with fluorescently conjugated secondary antibodies for 1.5 h at room temperature.

### 2.9. Flow Cytometry to Detect Cellular Chemoresistance

To explore cellular chemoresistance, a co-culture system was established using green fluorescence protein (GFP)-labeled lentiviral vectors. Wild-type cells were co-cultured with their lentiviral transduced counterparts (knockdown, overexpression, or empty vector control) at a 1:1 ratio in medium containing different concentrations (0–5 μg/mL) of cisplatin. Recombinant lentiviral constructs contain constitutive GFP expression, allowing quantitative tracking of the proportion of GFP+ cells by flow cytometric analysis.

### 2.10. Reactive Oxygen Species (ROS) and Antioxidant Capacity Detection

Intracellular ROS levels were assayed using an ROS reactive oxygen assay kit (Solarbio, Beijing, China, CA1420) according to the manufacturer’s instructions.

### 2.11. Measurement of Lipid Peroxidation Levels

Malondialdehyde (MDA) content was quantified in cell lysates using a commercial detection kit (Beyotime, Shanghai, China, S0131) according to the manufacturer’s instructions.

### 2.12. Determination of Intracellular Divalent Iron Ions

Fe^2^⁺ levels were detected using a FerroOrange probe (Dojindo Laboratories, Beijing, China, F374) according to the manufacturer’s instructions.

### 2.13. Mitochondrial Membrane Potential Assay

The mitochondrial membrane potential was measured using tetramethylrhodamine ethyl ester (TMRE) staining (mitochondrial membrane potential assay kit, Beyotime, Shanghai, China, C2001S) according to the manufacturer’s protocol.

### 2.14. Statistical Analysis

Data are presented as means ± SD unless otherwise noted. Comparisons used two-tailed Student’s *t*-tests. Survival analysis employed Kaplan–Meier curves with log-rank tests. A *p* value of <0.05 was considered significant.

## 3. Results

### 3.1. Elevated OLFM4 Expression in Cisplatin-Resistant HNSCC-PDO Models

**Figure 1:**
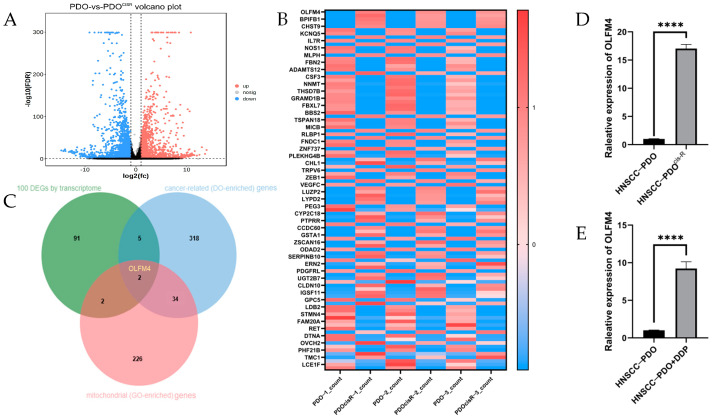
OLFM4 expression was significantly elevated in cisplatin-resistant HNSCC-PDOs (HNSCC-PDO^cis-R^). (**A**) Volcano plot of DEGs based on transcriptome sequencing using HNSCC-PDOs and HNSCC-PDO^cis-R^; (**B**) heatmap of the top 100 DEGs by transcriptome sequencing; (**C**) mapping of Wayne plots to screen out OLFM4 as a candidate gene among the top 100 DEGs identified by transcriptome sequencing; (**D**) data of qRT-PCR showing that OLFM4 expression was significantly upregulated in HNSCC-PDO^cis-R^; (**E**) qRT-PCR showed that OLFM4 in HNSCC-PDOs was significantly upregulated when treated with cisplatin. Note: DEGs, differentially expressed genes; HNSCC, head and neck squamous cell carcinoma; PDOs, patient-derived organoids; PDO^cis-R^, cisplatin-resistant PDOs. Notes **** *p* < 0.0001.

Analysis of RNA-seq data from our established HNSCC-PDO model and its cisplatin-resistant counterpart (HNSCC-PDO^cis-R^) revealed significant differential gene expression patterns associated with tumor progression and drug resistance. Through comprehensive bioinformatic analysis including volcano plots (Figure 1A), heatmaps of the top 100 DEGs (Figure 1B), and Venn diagram analysis of the top 100 DEGs and cancer-related (DO-enriched) and mitochondrial (GO-enriched) genes, OLFM4 emerged as a prime candidate for functional investigation (Figure 1C). qRT-PCR validation demonstrated a 16.03 ± 0.4328-fold increase in OLFM4 expression in HNSCC-PDO^cis-R^ compared to parental PDOs (Figure 1D). Notably, cisplatin treatment (3 μg/mL) induced 8.229 ± 0.5217-fold upregulation of OLFM4 in treatment-naïve HNSCC-PDOs (Figure 1E), suggesting its potential role in cisplatin-resistance mechanisms.

### 3.2. Clinical Correlation of OLFM4 Expression

**Figure 2:**
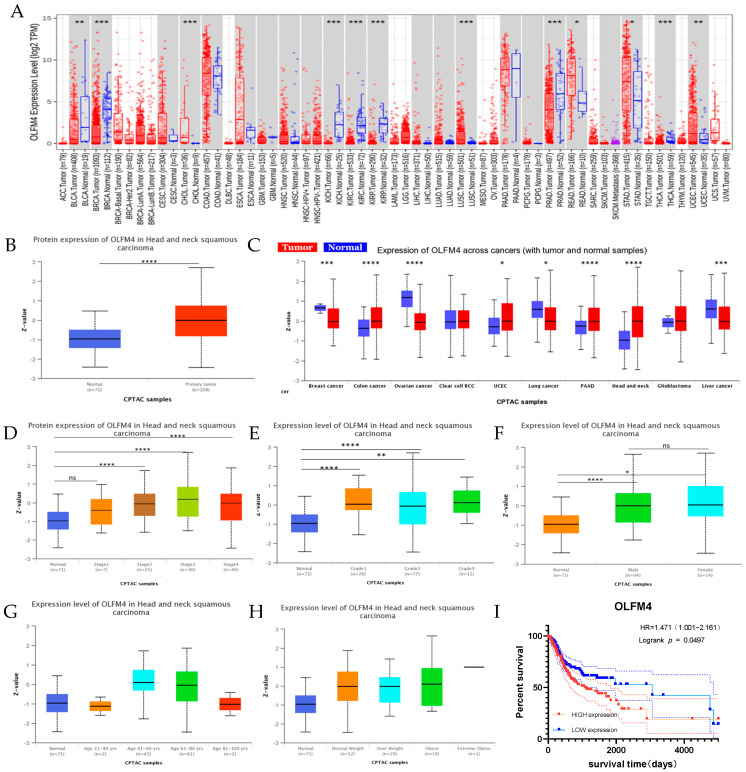
Correlations of clinical and pathological features with OLFM4. (**A**) Differential expression of OLFM4 in pan-cancer samples from TCGA database; (**B**) upregulated expression of OLFM4 in unpaired HNSCC samples from CPTAC database; (**C**) differential expression of OLFM4 in pan-cancer samples from CPTAC database; (**D**) OLFM4 expression in normal versus staged HNSCC tissues (stage I, stage II, stage III, and stage IV) based on CPTAC data; (**E**) OLFM4 expression in normal versus pathologically graded HNSCC (grade 1, grade 2, and grade 3) based on CPTAC data; (**F**) association between OLFM4 expression and gender in HNSCC based on CPTAC data; (**G**) association between OLFM4 expression and age in HNSCC based on CPTAC data; (**H**) association between OLFM4 expression and body weight in HNSCC based on CPTAC data; (**I**) overall survival of patients with HNSCC stratified by OLFM4 expression level (high or low) based on TCGA database (*p* = 0.0497). Notes: CPTAC, the Cancer Proteome Atlas Consortium; TCGA, The Cancer Genome Atlas; * *p* < 0.05, ** *p* < 0.01, *** *p* < 0.001; **** *p* < 0.0001.

TCGA data showed modest OLFM4 elevation in HNSCC versus normal tissues (*p* > 0.05, Figure 2A), while CPTAC analysis revealed significant upregulation (Figure 2B). Proteomic data identified consistent OLFM4 overexpression across multiple malignancies (Figure 2C), with significant stage- and grade-dependent increases in HNSCC (Figure 2D,E). Gender-specific analysis confirmed elevated OLFM4 in both male and female patients versus controls (Figure 2F), though no significant correlation with age or body weight was observed (Figure 2G,H). Critically, high OLFM4 expression correlated with poorer overall survival (OS) of HNSCC (HR = 1.471, *p* = 0.0497; Figure 2I).

### 3.3. Functional Characterization of OLFM4 in HNSCC

**Figure 3:**
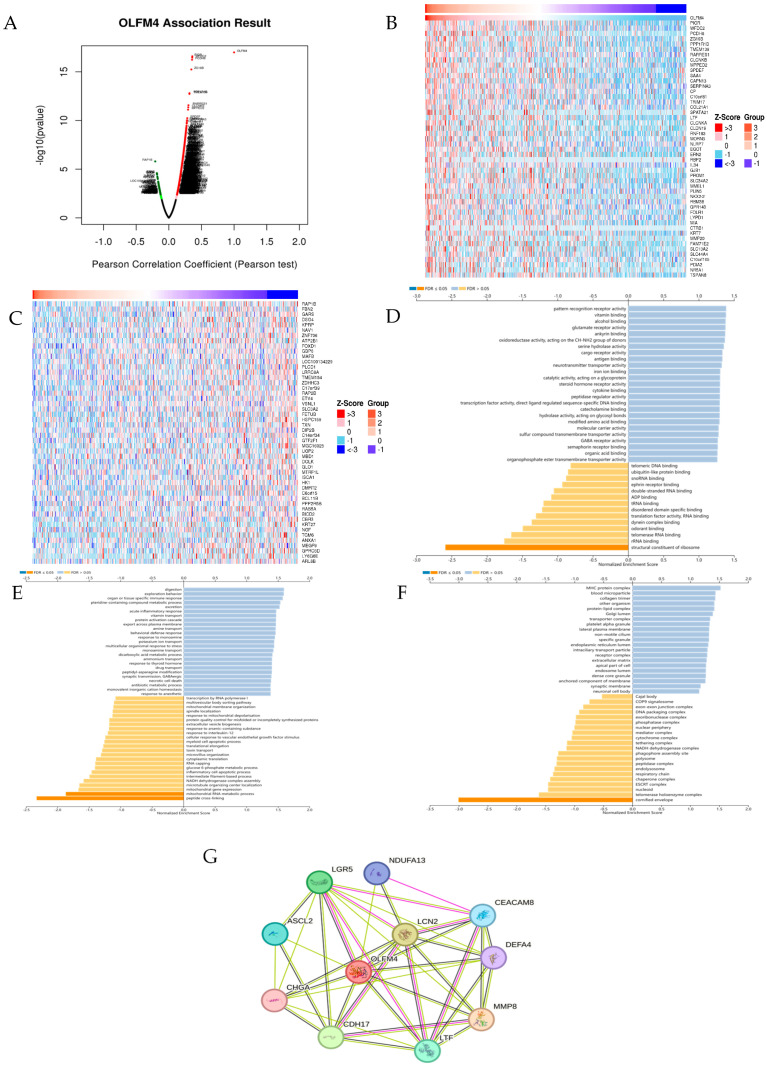
Predicted function of OLFM4 in head and neck squamous carcinoma (HNSCC). (**A**) Genes significantly related to OLFM4 expression; (**B**) top 50 genes significantly and positively related to OLFM4 expression; (**C**) top 50 genes significantly and negatively related to OLFM4 expression; (**D**) analysis of GO−enriched cellular components of genes significantly related to OLFM4; (**E**) analysis of GO−enriched biological processes of genes significantly related to OLFM4; (**F**) analysis of GO−enriched molecular functions of genes significantly related to OLFM4; (**G**) OLFM4 protein interaction network.

LinkedOmics co-expression analysis identified 6508 (red) positively and 13,656 negatively correlated genes (green) of OLFM4 (Figure 3A), with the top 50 associations visualized in heatmaps (Figure 3B,C). GO enrichment implicated OLFM4 in keratinization, mitochondrial respiration, function of NADH dehydrogenase, peptide cross-linking, mitochondrial RNA metabolism, ribosomal structure, rRNA binding, and redox enzyme activity (Figure 3D–F). STRING network analysis revealed protein partners (NDUFA13, MMP8, LTF, LCN2, LGR5, etc.) involved in mitochondrial respiration, iron transport, and neutrophil activation (Figure 3G), highlighting OLFM4’s multifunctional role in HNSCC development.

### 3.4. OLFM4 Promotes Aggressive HNSCC Phenotypes

**Figure 4:**
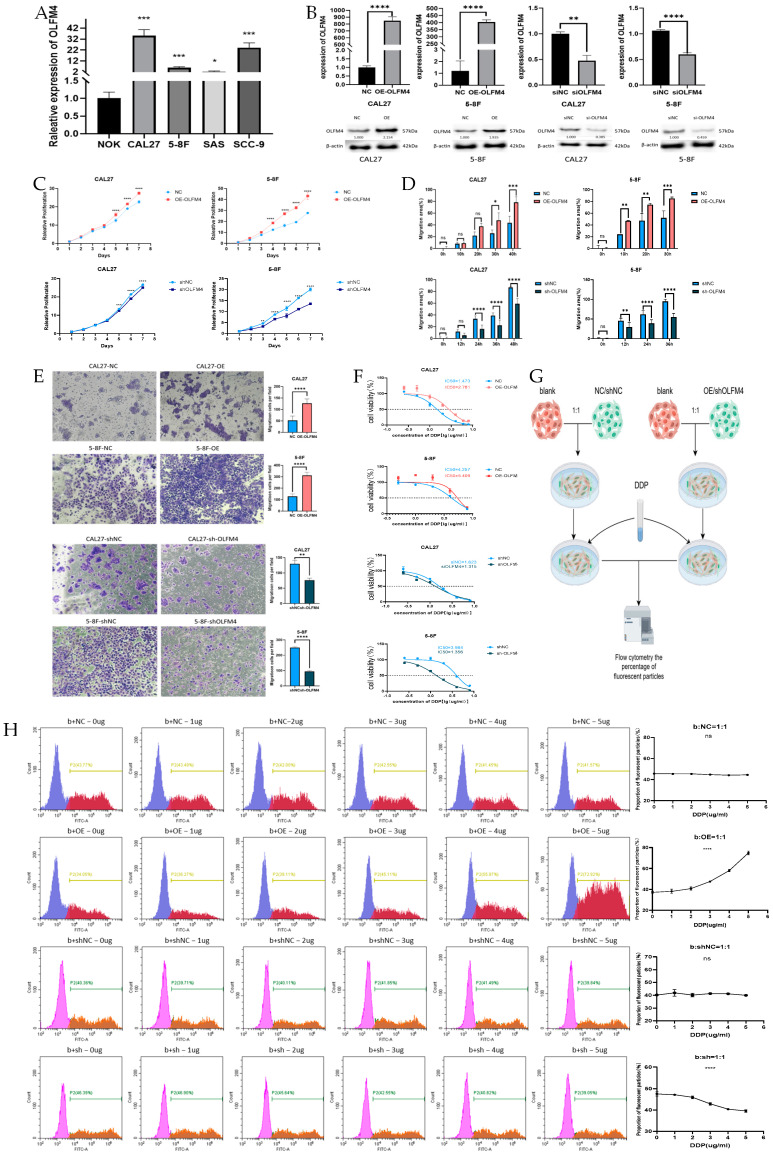
OLFM4 promoted proliferation, invasion, migration, and cisplatin resistance in HNSCC. (**A**) Expression of OLFM4 in different HNSCC cell lines and a normal cell line; (**B**) qRT-PCR and WB evaluating lentiviral transfection efficiency in CAL27 and 5-8F cells with overexpressed or knocked-down OLFM4; (**C**) association of OLFM4 expression with proliferation ability of CAL27 and 5-8F cells (CCK8 assay); (**D**) association of OLFM4 expression with migration ability of CAL27 and 5-8F cells (scratch assay); (**E**) association of OLFM4 expression with invasion ability of CAL27 and 5-8F cells (transwell assay); (**F**) measurements of IC50 of cisplatin in CAL27 and 5-8F cells; (**G**) experimental steps for the co-culture of GFP-labeled 5-8F cells of each group (5-8F-OE-OLFM4, 5-8F-shOLFM4, 5-8F-OE-NC, 5-8F-shNC cells) with wild-type 5-8F cells (blank control without GFP-labeling), respectively; (**H**) fluorescence intensity detected by flow cytometry in 5-8F-OE-OLFM4 cells co-cultured with wild-type 5-8F cells and 5-8F-shOLFM4 cells co-cultured with wild-type 5-8F cells under cisplatin pressure at different concentrations. Notes: b, blank control without GFP labeling; OE, overexpression; NC, overexpression negative control; sh, knockdown; shNC, knockdown negative control; GFP, green fluorescent protein; * *p* < 0.05, ** *p* < 0.01, *** *p* < 0.001; **** *p* < 0.0001.

Figure 4A demonstrates that OLFM4 expression was significantly lower in NOK cells compared to all tested HNSCC cell lines (CAL27, 5-8F, SAS, and SCC-9). qRT-PCR analysis confirmed successful genetic manipulation, showing: (i) significant OLFM4 upregulation in CAL27-OE-OLFM4 (847.9 ± 33.14-fold) and 5-8F-OE-OLFM4 cells (403.4 ± 8.864-fold), and (ii) marked downregulation in CAL27-shOLFM4 (0.5254 ± 0.0647-fold) and 5-8F-shOLFM4 cells (0.4633 ± 0.02235-fold) relative to their respective controls (Figure 4B). Western blot analysis corroborated these findings at the protein level (Figure 4B and Figure S1), validating the successful establishment of OLFM4-overexpressing and -knockdown models for subsequent functional studies. Functional characterization revealed that OLFM4 overexpression significantly enhanced proliferative capacity in both CAL27-OE-OLFM4 and 5-8F-OE-OLFM4 cells compared to controls (CCK-8 assay; Figure 4C), while OLFM4 knockdown produced the opposite effect. Consistently with these findings, wound healing assays showed accelerated migration in CAL27-OE-OLFM4 and 5-8F-OE-OLFM4 cells, but impaired migration in both CAL27-shOLFM4 and 5-8F-shOLFM4 cells (Figure 4D and Figure S2). Transwell invasion assays demonstrated increased invasive potential in CAL27-OE-OLFM4 and 5-8F-OE-OLFM4 cells versus suppressed invasion in knockdown cells (CAL27-shOLFM4 and 5-8F-shOLFM4 cells) (Figure 4E).

### 3.5. OLFM4 Promoted Cisplatin Resistance of HNSCC Cells

Cisplatin toxicity assays revealed distinct resistance patterns: CAL27-OE-OLFM4 (IC50 = 2.781 μg/mL) and 5-8F-OE-OLFM4 cells (IC50 = 5.409 μg/mL) demonstrated significantly enhanced cisplatin resistance compared to CAL27-OE-NC (IC50 = 1.473 μg/mL) and 5-8F-OE-NC (IC50 = 4.257 μg/mL), respectively. Conversely, OLFM4-knockdown cells CAL27-shOLFM4 (IC50 = 1.315 μg/mL) and 5-8F-shOLFM4 (IC50 = 1.356 μg/mL) showed markedly increased cisplatin sensitivity relative to their controls (CAL27-shOLFM4-NC (IC50 = 1.623 μg/mL) and 5-8F-shOLFM4-NC (IC50 = 3.984 μg/mL)), establishing OLFM4 as a key mediator of chemoresistance in HNSCC (Figure 4F). Notably, after successful lentiviral transfection, cells exhibited stable growth and normal morphology, with no significant signs of toxicity or abnormal cell death, ensuring that the observed effects on cisplatin resistance were directly attributable to OLFM4 manipulation rather than artifacts from the lentiviral infection process.

**Figure 5:**
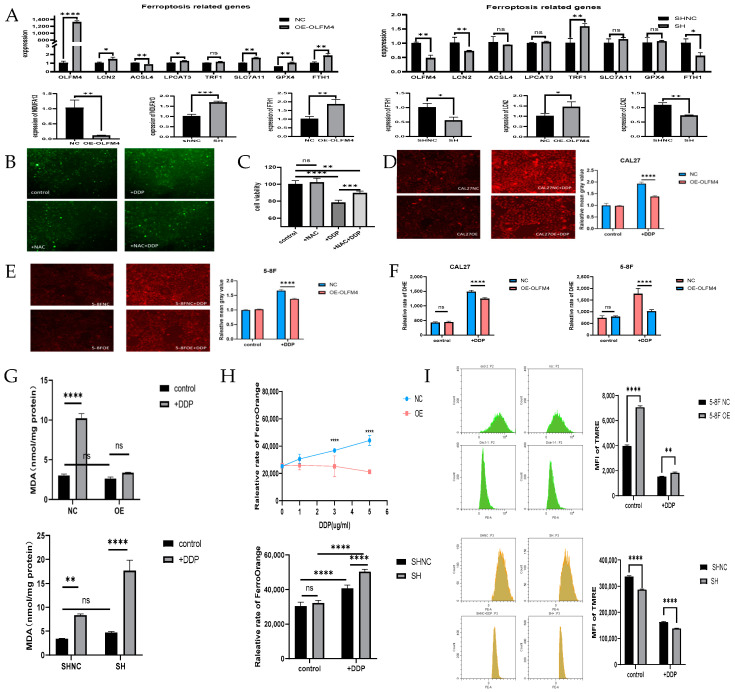
Effects of OLFM4 on cisplatin-resistance in HNSCC via mediation of ferroptosis, mitochondrial function, and accumulation of ROS and MDA. (**A**) qRT-PCR detection of OLFM4 affecting iron death and mitochondrial respiratory chain-related genes; (**B**) ROS staining shows that cisplatin can increase the level of cellular oxidative stress and is back-complemented by the ROS inhibitor NAC (N-acetyl-L-cysteine); (**C**) CCK8 shows that cisplatin decreases cellular activity and can be overcome by the ROS inhibitor NAC; (**D**,**E**) fluorescent staining verifying that OLFM4 expression level affected cisplatin-induced generation of ROS levels; (**F**) fluorescence zymography detection of OLFM4 expression levels affecting ROS levels induced by cisplatin; (**G**) OLFM4 expression levels affected MDA levels; (**H**) OLFM4 expression levels were related to intracellular ferrous ion content; (**I**) OLFM4 expression levels were related to mitochondrial membrane potential. Notes: DHE, dihydroethidium, an ROS indicator; MDA, malondialdehyde, indicator of lipid peroxidation; NAC, N-acetyl-L-cysteine, an inhibitor of ROS. * *p* < 0.05, ** *p* < 0.01, *** *p* < 0.001; **** *p* < 0.0001.

It was found that the relative change in IC50 values affected by OLFM4 expression levels was greater in 5-8F cells. This was followed up with further validation of the effect of OLFM4 on resistance-related metrics using 5-8F cells. To further validate these findings, this study employed a GFP-based competitive co-culture system. GFP-labeled experimental cells (5-8F-OE-OLFM4, 5-8F-OE-NC, 5-8F-shOLFM4, and 5-8F-shNC) were co-cultured with unlabeled control counterparts and exposed to escalating cisplatin concentrations (0, 1, 2, 3, 4, and 5 μg/mL). Strikingly, the proportion of GFP-positive OLFM4-overexpressing cells progressively increased with cisplatin concentration (Figure 5H), while GFP-labeled shOLFM4 cells exhibited dose-dependent depletion (Figure 5I). These results demonstrate that OLFM4 significantly enhances cisplatin resistance in HNSCC cells and may serve as a promising target for overcoming cisplatin resistance.

### 3.6. OLFM4 Modulates Mitochondrial Function- and Ferroptosis-Related Pathways in HNSCC Cells

Through integrated bioinformatic analysis and literature review, we identified key mitochondrial function- and ferroptosis-related genes (*LCN2*, *FTH1*, *ACSL4*, *TRF1*, *LPCAT3*, *SLC7A11*, *GPX4*, and *NDUFA13*) [20,21] potentially regulated by OLFM4. qRT-PCR analysis demonstrated significant alterations in their expression patterns: (i) marked upregulation of *LCN2*, *FTH1*, *LPCAT3*, *SLC7A11*, and *GPX4* (*p* < 0.05) and significant downregulation of *ACSL4* and *NDUFA13* (*p* < 0.01) in OLFM4-overexpressing cells (5-8F-OE-OLFM4 cells) versus their respective controls (Figure 5A); (ii) significant downregulation of *LCN2*, *FTH1*, and *TRF1* (*p* < 0.05) and notable upregulation of *NDUFA13* (*p* < 0.001) in OLFM4-knockdown cells (5-8F-shOLFM4 cells) versus their respective controls (Figure 5A). These findings reveal potential mechanistic links between OLFM4 and mitochondrial/ferroptosis pathways in HNSCC cells.

To further explore the functional implications of these genetic changes, the role of OLFM4 in oxidative stress regulation, ferroptosis, and mitochondrial function were investigated. Cisplatin-induced cytotoxicity was confirmed through ROS quantification and cell viability assays (Figure 5B,C). The results revealed that CAL27-OE-OLFM4 and 5-8F-OE-OLFM4 cells exhibited significantly reduced ROS accumulation compared to their respective controls under 2 μg/mL cisplatin treatment (*p* < 0.001, Figure 5D–F). This antioxidant effect of OLFM4 was consistently observed across both fluorescence microscopy and quantitative enzymatic assays. To evaluate lipid peroxidation regulation by OLFM4, MDA levels were investigated. As shown in Figure 5G, MDA levels were significantly lower in 5-8F-OE-OLFM4 cells and significantly higher in 5-8F-shOLFM4 cells compared to their respective controls.

Intracellular Fe^2^⁺ levels were also affected by OLFM4. In 5-8F-OE-OLFM4 cells, Fe^2^⁺ levels were significantly reduced compared to control cells (5-8F-OE-NC) following 2 μg/mL cisplatin treatment (*p* < 0.0001), while Fe^2^⁺ accumulation was markedly elevated in cisplatin-exposed 5-8F-shOLFM4 cells relative to their controls (5-8F-shNC cells) (*p* < 0.0001) (Figure 5H).

TMRE assays revealed that the mitochondrial membrane potential (ΔΨm) significantly increased in 5-8F-OE-OLFM4 cells compared to control cells (5-8F-OE-NC), whereas 5-8F-shOLFM4 cells exhibited a significantly reduced ΔΨm relative to their controls (5-8F-shNC) when treated with 2 μg/mL cisplatin (Figure 5I). These collective findings strongly suggest that OLFM4 modulates mitochondrial function- and ferroptosis-related pathways in HNSCC cells, potentially contributing to cisplatin resistance.

## 4. Discussion

Our study elucidates the multifaceted role of OLFM4 in HNSCC progression and chemoresistance. A an extracellular matrix glycoprotein containing distinctive N-linked carbohydrate chains and olfactomedin-like domains, OLFM4 has emerged as a critical regulator of oxidative stress responses and tumor progression [22,23,24,25,26]. The current findings demonstrate that OLFM4 not only drives aggressive tumor behavior but also mediates cisplatin resistance through novel molecular mechanisms, positioning it as a promising therapeutic target for HNSCC.

The anti-apoptotic properties of OLFM4 under oxidative stress conditions, particularly its ability to protect tumor cells from hydrogen peroxide-induced damage [22], may explain its crucial role in treatment resistance. Our observations align with previous reports documenting OLFM4’s involvement in cell cycle regulation, specifically its promotion of S- to G2/M-phase transition through growth inhibition mechanisms [27,28]. While OLFM4’s oncogenic functions have been characterized in various malignancies, including its established role as a metastasis marker in ER+ breast cancer [29] and its association with cancer stem cell properties in hepatocellular carcinoma [30], its specific contributions to HNSCC pathogenesis remain underexplored. The present study addresses this knowledge gap by demonstrating OLFM4’s significant correlation with poor OS in HNSCC patients from a cohort from TCGA, along with its functional promotion of invasion, migration, and proliferation in CAL27 and 5-8F cell lines.

RNA-seq and bioinformatic analysis in this study link OLFM4 overexpression to HNSCC progression and poor prognosis, supported by functional evidence of its role in enhancing proliferation, invasion, and cisplatin resistance. While TCGA mRNA data showed only modest OLFM4 upregulation, the significant protein-level elevation in CPTAC datasets highlights potential post-translational regulation, a critical consideration for therapeutic development. The stage/grade-dependent OLFM4 increase and survival correlation further validate its clinical utility as a prognostic biomarker.

The chemoresistance mechanisms were identified to operate through two parallel pathways. First, LCN2/FTH1 upregulation implies enhanced iron storage/handling, and OLFM4 appears to suppress ferroptosis by upregulating key mediators (FTH1 and LCN2) and reducing Fe^2^⁺ accumulation, thereby inhibiting ferroptosis pathway and limiting lipid peroxidation [31]. This finding is particularly significant given the established role of Fe^2^⁺ in promoting ROS generation through Fenton reactions, which exacerbate lipid peroxidation, leading to damage of the membrane integrity and death of the tumor cells [32]. Increased expression of GPX4, a key enzyme in the glutathione peroxidase system, directly inhibits ferroptosis by reducing lipid peroxide levels [33]. Conversely, the downregulation of ACSL4, which is involved in the synthesis of lipid peroxides required for ferroptosis initiation, further supports the inhibitory effect on ferroptosis [34]. Second, OLFM4 enhances mitochondrial stability through ΔΨm elevation and NDUFA13 subunit suppression, resulting in apoptosis resistance. It was suggested that downregulation of NDUFA13, which is a subunit of mitochondrial complex I, reduces ROS production and promotes resistance of cancer cells to apoptosis [35,36]. These dual mechanisms provide a comprehensive explanation for OLFM4-mediated cisplatin resistance in HNSCC.

Several limitations should be acknowledged. First, although our PDO model provides valuable insights, the specific molecular interactions between OLFM4 and proteins involved in ferroptosis and mitochondrial function demand validation via co-immunoprecipitation (co-IP). Future co-IP studies will explore potential associations between OLFM4 and key proteins in the ferroptosis pathway, such as LCN2 and FTH1, to clarify the underlying mechanisms. Second, the clinical significance of our findings requires confirmation through studies using larger clinical cohorts. A prospective design will enable systematic collection of clinical data, including treatment responses and long-term outcomes, from HNSCC patients. This approach will facilitate more accurate assessment of the predictive value of OLFM4 expression levels for cisplatin resistance and patient survival. Third, the possible cross-talk between OLFM4-mediated resistance pathways and tumor microenvironment (TME) factors merits further investigation. Future research will focus on exploring how OLFM4 affects the interactions between HNSCC cells and immune cells, such as tumor-associated macrophages and T lymphocytes, which are the main components in TME and are involved in tumor ferroptosis and chemoresistance [37].

Despite these limitations, our study represents the first comprehensive evaluation of OLFM4’s role in HNSCC using cisplatin-resistant PDO models. The consistent results across molecular, cellular, and clinical analyses strongly support OLFM4’s potential as both a prognostic biomarker and therapeutic target. Future studies should explore OLFM4-targeted strategies, particularly in combination with existing cisplatin regimens, to overcome treatment resistance in advanced HNSCC cases.

## 5. Conclusions

In summary, our findings establish OLFM4 as a critical oncogenic driver in HNSCC progression and prognosis, demonstrating that its upregulation promotes tumor aggressiveness through enhanced proliferation, migratory, and invasive capacities while simultaneously conferring cisplatin resistance. The strong correlation between OLFM4 expression and poor clinical outcomes highlights its dual role as both a prognostic biomarker and a promising therapeutic target for HNSCC treatment. These results provide a compelling rationale for future development of OLFM4-targeted therapies to improve clinical management of this malignancy.

## Figures and Tables

**Table 1:** Primer sequences.

Gene	Primer Sequences (5′ → 3′)
GAPDH-F	TGCAACCGGGAAGGAAATGA
GAPDH-R	GTGGAATTTGCCATGGGTGGA
OLFM4-F	CATCTGCTTCTAACGCCTTCAT
OLFM4-R	TAGTTTGCCCTCTTTCCCTGTG
LCN2-F	CCCCATCTCTGCTCACTGTC
LCN2-R	TTTTTCTGGACCGCATTG
ACSL4-F	GCTACTTGCCTTTGGCTCATGTGC
ACSL4-R	GTGTGGGCTTCAGTACAGTACAGTCTCC
LPCAT3-F	TCAGGATACCTGATTTGCTTCCA
LPCAT3-R	GGATGGGTCTGTTGCACCAAGTAG
TfR1-F	ACCATTGTCATATACCCGGTTCA
TfR1-R	GGCCTTTGTGTTATTGTCAGCAT
SLC7A11-F	TGCTGGGCTGATTTTATCTTCG
SLC7A11-R	GAAAGGGCAACCATGAAGAGG
GPX4-F	GAAGCAGGAGCCAGGGAGTA
GPX4-R	GGTGAAGTTCCACTTGATGGC
FTH1-F	AATTTCTTGACCCACTGGTGCACT
FTH1-R	TCGAATCGAGAGTAGTGGCACA
NDUFA13-F	CTTGATCGGGGAGCTGTACG
NDUFA13-R	GGCCTACGTGTACCACATGA

## Data Availability

The data that support the findings of this study are available from the corresponding author upon reasonable request.

## Supplementary Materials

The following supporting information can be downloaded at https://www.mdpi.com/article/10.3390/curroncol32050276/s1. Figure S1: The whole blot showing all the bands with all molecular weight markers; Figure S2: Supplementary images of the cell migration experiment.

## Abbreviations

The following abbreviations are used in this manuscript:

HNSCC	head and neck squamous carcinoma
DEGs	differentially expressed genes
PDO	patient-derived organoid
PDO^cis-R^	patient-derived organoid cisplatin-resistant counterpart
OLFM4	olfactomedin 4
