# Supplementary material for: The Association of OLFM4 with the Progression and Cisplatin Resistance of Head and Neck Squamous Carcinoma
Source: Curr Oncol. 2025 May 13;32(5):276. doi: 10.3390/curroncol32050276 (PMC12110400; doi:10.3390/curroncol32050276)
Supplement: Supplementary file 1 [file curroncol-32-00276-s001.zip › curroncol-3613913-supplementary.pdf]

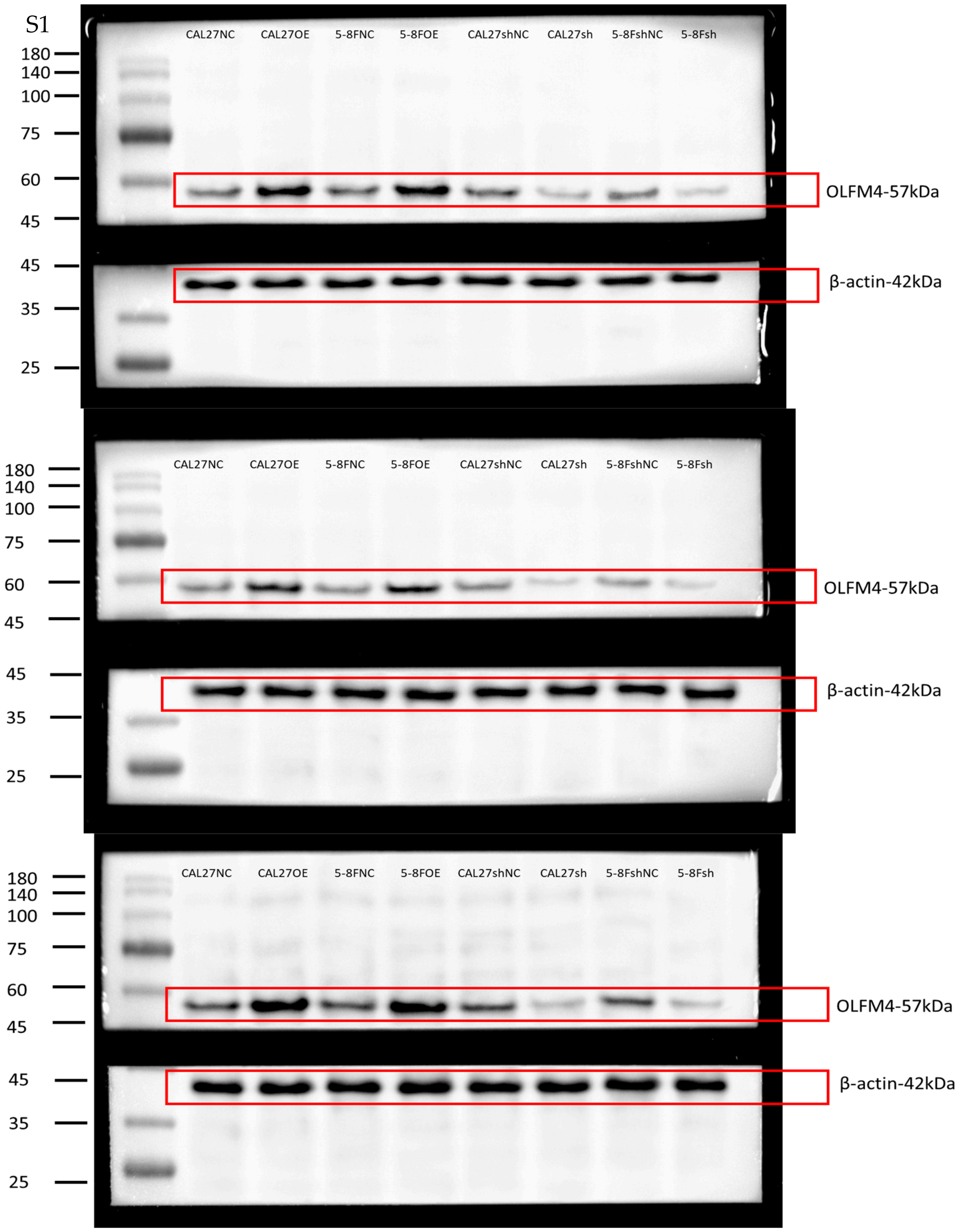

Figure S1. The whole blot showing all the bands with all molecular weight markers.

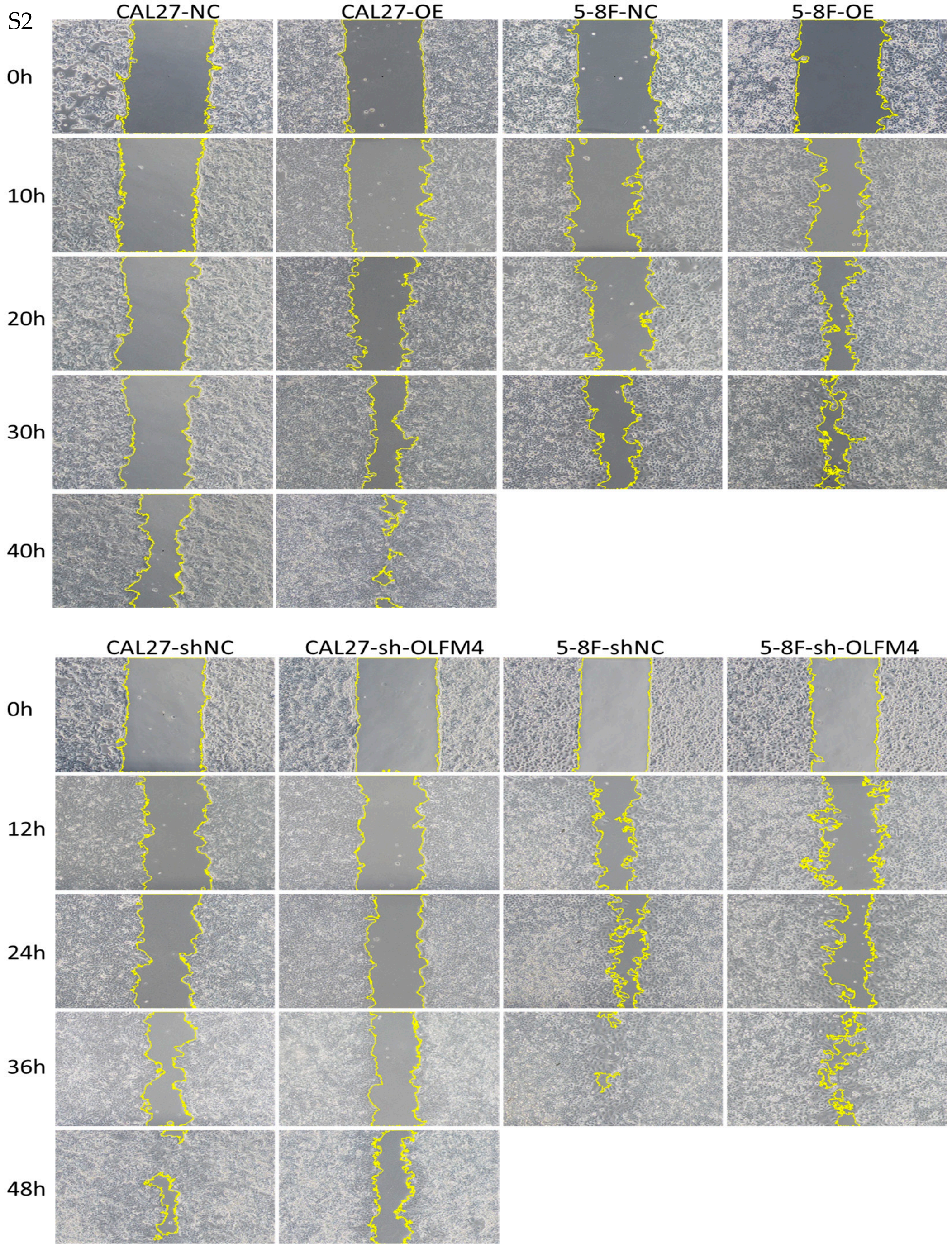

Figure S2. Supplementary images of the cell migration experiment.
